# Anti-inflammatory treatment after selective laser trabeculoplasty: a systematic review of the literature and meta-analysis of randomized control trials

**DOI:** 10.5935/0004-2749.2021-0353

**Published:** 2023

**Authors:** Dervenis Panagiotis, Dervenis Nikolaos, Chiras Dimitrios, Vasilakis Panagiotis

**Affiliations:** 1 School of Medicine, University of Thessaly, Larissa, Greece; 2 Department of Ophthalmology, General Hospital of Trikala, Trikala, Greece; 3 St Paul’s Eye Unit, Royal Liverpool University Hospital, Liverpool, United Kingdom; 4 Department of Ophthalmology, Famagusta General Hospital, Paralimni, Cyprus

**Keywords:** Glaucoma, Trabeculectomy, Laser therapy, Anterior chamber, Trabecular meshwork, Anti-inflammatory agents, non-steroidal, Intraocular pressure, Randomized controlled trials as topic, Glaucoma, Trabeculectomia, Terapia a laser, Câmara anterior, Malha trabecular, Anti-inflamatórios não esteroides, Pressão intraocular, Ensaios clínicos controlados aleatórios como assunto

## Abstract

We assessed the effects of anti-inflammatory treatment after selective laser trabeculoplasty through a systematic search of the MEDLINE, COCHRANE, and ClinicalTrials.gov. The outcome measures were intraocular pressure, anterior chamber inflammation, and discomfort. Evidence synthesis was performed using fixed effects or random-effects model according to the heterogeneity of the included studies. Heterogeneity was assessed using Q-statistic and I^2^. For an overall estimate of continuous outcomes, the mean differences and their 95% confidence intervals were applied, while odds ratios and their 95% confidence intervals were applied for dichotomous outcomes. Six studies were included in all. No significant difference was noted in the patients for intraocular pressure and discomfort when treated with anti-inflammatory drops. However, the patients showed benefit from reduced anterior chamber inflammation in the first postoperative week [FE OR=0.43, 95% CI=(0.19, 0.95), P_Q_=0.97, I^2^=0%], with no significant difference between the outcomes of non-steroidal anti-inflammatory drugs and steroids [FE OR=0.75, 95% CI=(0.20, 2.82), P_Q_=0.37, I^2^=0%]. Anti-inflammatory drops reduce anterior chamber inflammation after selective laser trabeculoplasty but showed no effect on the intraocular pressure.

## Abstract (pt)

O objetivo deste estudo é avaliar os efeitos do tratamento anti-inflamatório após a trabeculoplastia seletiva a laser. Uma busca sistemática foi feita no *MEDLINE, COCHRANE* e *ClinicalTrials.gov*. As medidas de resultado foram pressão intraocular, inflamação da câmara anterior e desconforto. A síntese de evidência foi realizada utilizando-se modelo de efeitos fixos ou efeitos aleatórios, de acordo com a heterogeneidade dos estudos incluídos. A heterogeneidade foi avaliada utilizando-se Q-statistic e I². Para uma estimativa global dos resultados contínuos, foram usadas diferenças médias e seus intervalos de confiança de 95% enquanto para resultados dicótomos, usou-se odds ratios e seus intervalos de confiança de 95%. Seis estudos foram incluídos. Nenhuma diferença significativa foi encontrada em pacientes tratados com gotas anti-inflamatórias em termos de pressão intraocular e desconforto. No entanto, eles se beneficiaram da redução da inflamação da câmara anterior na primeira semana pós-operatória [FE OR=0,43, IC 95% = (0,19, 0,95), P_Q_=0,97, I^2^=0%], sem diferença significativa entre anti-inflamatórios não esteroidais e esteroidais [FE OR=0,75, IC 95% = (0,20, 2,82), P_Q_=0,37, I^2^=0%]. Gotas anti-inflamatórias reduzem a inflamação da câmara anterior após trabeculoplastia seletiva a laser, não afetando a pressão intraocular.

## INTRODUCTION

Glaucoma is the leading cause of irreversible blindness across the world, with an estimated >70 million people suffering from all types of glaucoma globally, 10% of whom are bilaterally blind^(1)^. Owing to the possible asymptomatic nature of glaucoma until it becomes severe, it is hypothesized that the actual number of people affected is much higher than the number of people diagnosed with it^(2,3,4)^. Glaucoma is characterized by progressive degeneration of retinal ganglion cells that results in the cupping of the optic disk and visual loss. The main treatment options included the use of ocular hypotensive drops, laser trabeculoplasty, and different types of surgery to reduce intraocular pressure (IOP)^(5)^. Argon Laser Trabeculoplasty (ALT) provided IOP reduction by increasing the aqueous outflow^(6,7,8,9,10,11)^. It has been postulated that laser-induced thermal burns to the trabecular meshwork (TM) cause collagen and tissue contraction, which in turn reduces the diameter of the inner trabecular ring, reverses the collapse of the meshwork, and consequently maintains sufficient aqueous outflow^(7)^. ALT provides approximately a 30% reduction from the baseline IOP. The efficacy of ALT seemed to be related to pre-operative IOP, which makes ALT ineffective in eyes with Normal-Tension Glaucoma (NTG)^(6)^. While Pigmentary (PG) and Pseudoexfoliative Glaucoma (PEXG) exhibited a similar response to Primary Open Angle Glaucoma, patients suffering from PEXG seemed to benefit more from ALT^(12)^. ALT is not free of adverse effects; it has been reported to frequently cause IOP spikes following laser, development of peripheral anterior synechiae, corneal endothelial changes, and acute anterior uveitis (AAU). Owing to the thermal damage induced to the TM, its repeatability is also limited^(6)^. ALT is equivalent to a single topical medication as a primary treatment at 6 months and 1 year and 2 years after the treatment, but inferior at 5 years or when two topical medications were used^(13)^. ALT has also been shown to be inferior to trabeculectomy, with the latter achieving significantly lower IOPs and reduced diurnal fluctuation^(14)^.

Latina and Park introduced Selective Laser Trabeculoplasty (SLT) in 1995. SLT uses a 532-nm, Q-switched, frequency-doubled neodymium-doped yttrium aluminum garnet laser (Nd:YAG). Its application to the TM prevents heat dissipation outside the pigmented TM cells and reduces the collateral damage^(15)^. While SLT uses the same mechanism for reducing IOP by increasing the aqueous outflow through TM^(16,17)^, histopathological studies have reported less disruption to the TM in eyes post-SLT when compared to that post-ALT^(18)^. Various studies have suggested that the decrease in IOP may be attributed to an increase in the pro-inflammatory cytokine expression^(19)^, which induces an increase in the stromelysin-1 content that causes an increase in the aqueous outflow through the juxtacanalicular meshwork^(20)^. Furthermore, it has been postulated that TM monocyte activation after SLT increases the aqueous outflow *in vivo* and the Schlemm’s canal permeability *in vivo* either through cytokine secretion or through direct phagocytosing of debris in the TM^(21)^. Moreover, *in vivo* studies have reported that SLT and prostaglandin analogs (PGA) probably share a common action mechanism^(22)^. Therefore, inflammation may be the cornerstone in the efficacy of SLT and the appropriate use of anti-inflammatory agents after SLT is a current area of controversy.

No consensus statement exists regarding the postoperative management of patients after SLT. It remains debatable whether patients after SLT should use any drops they have been taking for a while. In SLT, it is common not to prescribe any steroids postoperatively, as these agents may blunt the biological effects of the laser. However, some surgeons prescribe a short course of anti-inflammatory medications to limit ocular discomfort, although this practice is not validated. The present study addresses the effect of post-SLT anti-inflammatory treatment in terms of efficacy and adverse events. Patients with various types of glaucoma were included in this meta-analysis considering that SLT can be used for different types of open-angle glaucoma. The main options for post-SLT anti-inflammatory treatment consist of either steroids or non-steroidal anti-inflammatory drops (NSAIDs). The present review evaluates the use of anti-inflammatory treatment in patients who underwent SLT in terms of IOP reduction, inflammation, and discomfort.

## METHODS

### Evidence acquisition

The present study was conducted in accordance with the PRISMA Statement guidelines^(23)^.

### Eligibility criteria

The studies included in our analysis met the following inclusion criteria:

- Publication before April 30, 2020;
- Designed as randomized control trials (RCT);
- Include at least one intervention group randomized to anti-inflammatory agents (NSAIDs or steroids) and another to placebo or no treatment;
- Includes numeric data for each time frame analyzed;
- involves subjects who either suffered from any type of open-angle glaucoma or ocular hypertension (OHT) and had undergone SLT.

The study exclusion criteria included the following:

- Reports not published in English;
- Conference abstracts;
- Pilot trials;
- Only graphically presented results;
- A statistically significant difference in the baseline IOP among groups;
- And retracted papers.

#### Search method

A meticulous literature search was conducted across the MEDLINE, COCHRANE, and ClinicalTrials.gov databases to identify all relevant RCTs from inception until the present. Furthermore, for the retrieved studies, a manual search was performed in their references to find possible past reports. The search strategy included the terms “anti-inflammat*”, “Non-Steroidal [MeSH Terms]”, “nsaid*”, “steroids [MeSH Terms]”, “laser trabe-culoplasty”, “selective laser trabeculoplasty”, and “SLT”. Specifically, for MEDLINE, the following search strategy using the Boolean Operators “OR” and “AND” was used:

(Anti-Inflammat*, Non-Steroidal[MeSH Terms]) OR (nsaid*) OR (steroids[MeSH Terms]) AND (laser trabeculoplasty OR SLT OR selective laser trabeculoplasty)

All titles and abstracts that were retrieved were reviewed for eligibility. For titles and abstracts of potentially eligible studies, the full texts were screened.

### Quality assessment

The Risk of Bias (RoB) Cochrane Tool for Systematic Reviews of Interventions was used to evaluate the retrieved RCTs^(24)^. RoB was used to assess several domains of bias considering the trial design, conduct, and reporting, as of low risk of bias, high risk of bias, or unclear risk of bias.

### Outcome measures

The primary outcome of the present study was the comparison of IOP in 1 week, 4-6 weeks, and 3-4 months after SLT between patients treated with anti-inflammatory drugs versus those who received placebo. Moreover, the secondary outcomes of the study were the presence of anterior chamber (AC) inflammation and patient discomfort after treatment between the study groups. The discomfort was defined as a feeling of pain, itching, burning, and a foreign body sensation.

### Data extraction

From the retrieved studies, the following data were extracted: author’s name, number of subjects enrolled, types of glaucoma analyzed, degrees of angle treated with SLT, energy used, baseline IOP, post-SLT interventions, dosage, outcomes measured, and study design. RoB assessment and data extraction were conducted by two authors (P.D .and N.D). In case of disagreement, a decision was made through consensus.

### Statistical analysis

Review Manager (RevMan [Computer program]. Version 5.3. Copenhagen: The Nordic Cochrane Centre, The Cochrane Collaboration, 2014) was used for all statistical analyses. Regardless of the study’s design, if one study included an examination of two different anti-inflammatory interventions, pooled measures were used to combine the results of these groups as suggested by the Cochrane Handbook for Systematic Reviews of Interventions guidelines^(25)^.

For continuous data, mean differences (MDs) were calculated for each time frame and their precision [95% confidence intervals (95% CIs)]. For binary outcomes, Odds Ratios (ORs) and their precision (95% CIs) were applied. Pooled estimates were calculated either with fixed effects (FE) or random effects (RE). The weight of each study was calculated as the inverse variance of individual effects. Considering that Q-statistic has low power when only a few studies are included^(26)^, heterogeneity among the studies was tested with both the Q-statistic and I^2^ tests^(27)^. Heterogeneity was assumed if P_Q_<0.1 or I^2^>50%. If significant heterogeneity was noted, the result was based on the RE model. Otherwise, the FE model was used. No publication bias was assessed because the number of retrieved studies was <10. However, communication was established with the study authors whenever possible to retrieve the missing data.

## RESULTS

### Study selection

**Figure 1:**
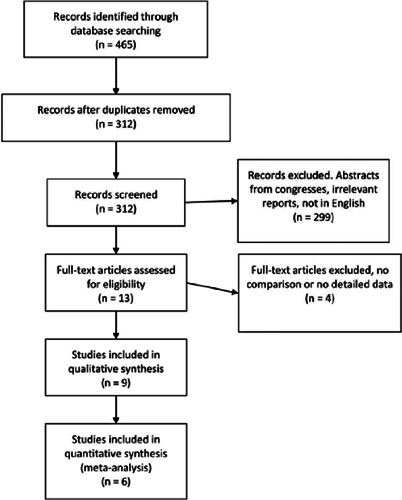
Flow chart depicting the literature search.

The flow diagram of the study selection is presented in figure 1. A literature search was performed on April 30, 2020. A total of 465 studies were identified from the database search. After removing any possible duplicates, 312 unique papers were found to be of our interest. We meticulously screened these records for relevance and retrieved 13 possible studies, followed by their full texts assessed to validate eligibility. Finally, 9 studies were included in our qualitative analyses^(28,29,30,31,32,33,34,35,36)^. Out of these 9 studies, 3 were excluded due to the lack of numerical results or because of a study design other than RCT^(31,35)^, such that a final of 6 studies were included in the meta-analysis. Whenever possible, communication was established with the authors to obtain more data from the published studies.

### Study characteristics and methodological quality assessment

**Figure 2:**
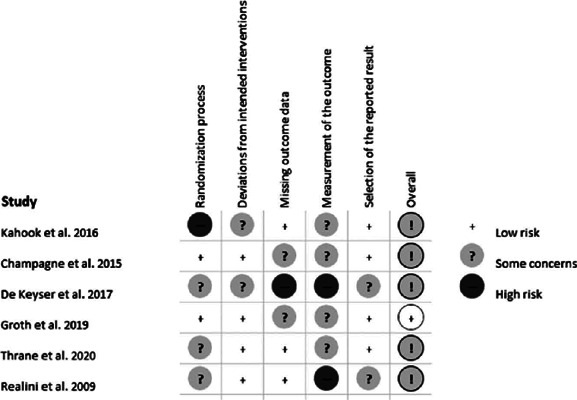
The summary of the risk of bias.

There were 4 studies that compared placebo or no treatment with both a steroid and an NSAID treatment. When required, steroid and NSAID groups were combined as described in the Methods section. One study included one arm of steroid treatment versus one arm of no treatment. One study compared an NSAID after SLT, a placebo group before and after SLT, and a group with apraclonidine pre-SLT and placebo post-SLT. The apraclonidine and placebo groups were also combined whenever needed. Details on the cases enrolled, types of glaucoma treated, degrees of TM treated, energy level used, baseline IOPs, outcomes measured, and the study design are presented in table 1. The quality of the studies included was assessed according to the RoB Cochrane tool for Systematic Reviews of interventions (Figure 2).

**Table 1:** Characteristics of the included studies

Study	Cases	Types of glaucoma	Degrees of SLT	Energy	Baseline IOP	Post SLT Interventions	Dosage	Outcomes	Study design
Realini et al.^(33)^ 2009	25	OAG	360°	100 pulses of mean power per shot 0.9 mJ	18.4±4.1 18.4±4.3	No treatment Prednisolone 1%	1 drop 4 times daily for 7 days	• IOP • AC inflammation	Prospective, observer blinded, RCT
Kahook^(32)^ 2016	31	OAG	No information	No information	17.8±4.5 17.0±3.3 16.1±3.1	Refresh Tears Prednisolone 1% Ketorolac 0.5%	1 drop 4 times daily for 4 days	IOP	Prospective, observer blinded RCT
Champagne et al.^(28)^ 2015	96	OAG	180°	50 pulses initially set at 0.7 mJ Total: Placebo: 43.60±2.01 Prednisolone: 45.20±1.86 Ketorolac: 48.04±1.82	25.01±0.47 25.86±0.69 25.85±0.70	Placebo Prednisolone 1% Diclofenac 0.1%	1 drop 4 times daily for 5 days	• IOP • Success of the SLT • AC inflammation	Prospective, double blinded RCT
De Keyser et al.^(29)^ 2017	132	• OAG • NTG • OHT	360°	No treatment: 1.09 Dexamethasone 0.1%: 1.11±0.35 Indomethacin 0.1%: 1.07±0.30	- 22.02 23.98	No treatment Dexamethasone 0.1% Indomethacin 0.1%	1 drop 3 times daily for 7 days	• IOP • IOP Spike • AC • Inflammation • Discomfort • Effect on efficacy	Prospective RCT
Growth et al.^(30)^ 2019	96	• OAG (75%) • PXG (4%) • Pigmentary (21%)	180° (36% 270 (12%) 360° (52%)	50 to 100 pulses set at 0.8 mJ	22.7±7 23.7±4.2 23.3±4.2	Placebo Prednisolone 1% Ketorolac 0.5%	1 drop 4 times daily for 5 days	• IOP • AC • Inflammation • Discomfort	Prospective, double blinded RCT
Thrane et al.^(34)^ 2020	50	• OAG (24%) • PXG (43%) • NTG (24%) • OHT (10%)	360°	110 pulses set at 0.3 to 1.2 mJ	17.3 19.3 17.2	Placebo Apraclonidine 10mg/mL Diclofenac 1mg/mL	1 drop 4 times daily for 5 days	• IOP • IOP Spike • AC • Inflammation • Discomfort	Prospective, double blinded RCT

IOP= Intraocular pressure.

OHT= Ocular Hypertension.

OAG= Open angle glaucoma.

PXG= Pseudoexfoliative glaucoma.

NTG= Normal-tension glaucoma.

AC= Anterior Chamber.

SLT= Selective Laser Trabeculoplasty.

RCT= Randomized Control Trial.

### A. Analysis per IOP

#### NSAIDs versus Steroids

**Figure 3:**
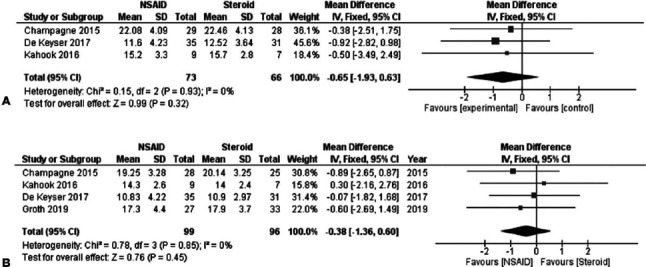
(A) IOP measurements 1 week after SLT-NSAID vs. Steroid. (B) IOP measurements 4-6 weeks after SLT-NSAID vs, Steroid.

Three studies including 139 subjects provided data for direct comparisons of the IOP 1 week after SLT. All these studies provided comparable baseline IOPs for both arms. The overall pooled difference between the 2 treatments after synthesizing the outcomes of the 3 studies did not reveal any statistically significant difference between the groups [FE MD=-0.65, 95% CI = (-1.93, 0.63), P_Q_=0.93, I^2^=0% (Figure 3A)].

In order to obtain an overall estimate regarding the IOPs 4-6 weeks after SLT and data from 4 studies were synthesized. A total of 195 subjects were included in this analysis. The overall pooled estimate for this comparison revealed that both the groups did not differ significantly [FE MD=-0.38, 95% CI = (-1.36, 0.60), P_Q_=0.85, I^2^=0% (Figure 3B)].

#### Anti-Infammatory versus Placebo/No treatment

For assessing the effects of anti-inflammatory treatments after SLT, we combined the data from studies assessing both NSAIDs and steroids versus placebo or no treatment, as described in the Methods section. The time frames examined included 1 week after SLT, 4-6 weeks after SLT, and 3-4 months after SLT. Baseline IOPs did not show a statistically significant difference between the treatment arms.

**Figure 4:**
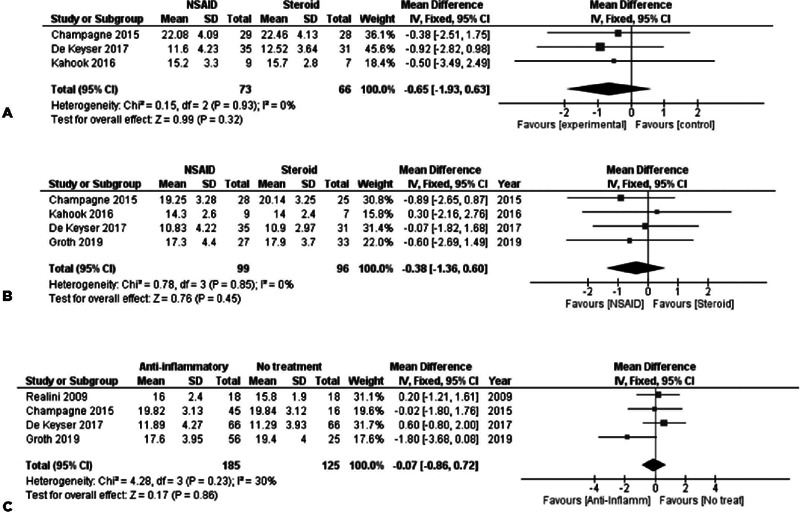
(A) IOP measurements 1 week after SLT-anti-inflammatory vs. No treatment. (B) IOP measurements 4-6 weeks after SLT-anti-inflammatory vs. No treatment. (C) IOP measurements 3-4 months after SLT-anti-inflammatory vs. No treatment.

The first postoperative week was assessed by synthesizing 5 studies with 344 subjects. The use of anti-inflammatory drugs post-SLT was not found to be associated with early IOP measurements [FE MD=0.16, 95% CI = (-0.65, 0.97), P_Q_=0.97, I^2^=0% (Figure 4A)].

After 4-6 weeks, the use of anti-inflammatory treatment was found to be associated with lower IOP measurements, without reaching any statistical significance levels. Six studies with 427 subjects participated in the synthesis, with a FE MD=−0.48, 95% CI = (−1.14, 0.18), P_Q_=0.53, I^2^=0% (Figure 4B).

In the last postoperative period 3-4 months after SLT, no association was identified between topical anti-inflammatory treatment and IOP. Four studies with 310 subjects were included in the synthesis, with studies presenting moderate heterogeneity [FE MD=-0.07, 95% CI = (-0.86, 0.72), P_Q_=0.23, I^2^=30% (Figure 4c)].

**Figure 5:**
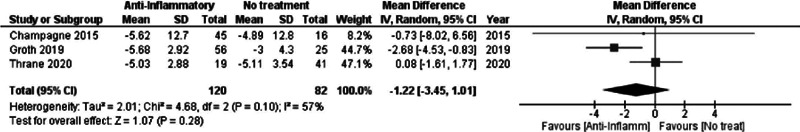
Mean reduction in IOP 12 weeks after SLT-anti-inflammatory vs. No treatment.

In order to compare the mean reduction in IOP 12 weeks after SLT, 3 studies were combined, including a total of 202 subjects. No clear benefit was exhibited from the use of anti-inflammatory treatment [RE MD=−1.22, 95% CI = (−3.45, 1.01), P_Q_=0.10, I^2^=57% (Figure 5)]. However, the included studies presented moderate heterogeneity.

### B. Analysis per AC Inflammation

In order to be robust about the effect of each treatment on the development of AC inflammation, only the presence or absence of AC activity was assessed, hence the treatment effect was expressed as a binary outcome.

#### 1. NSAIDs versus Steroids

**Figure 6:**
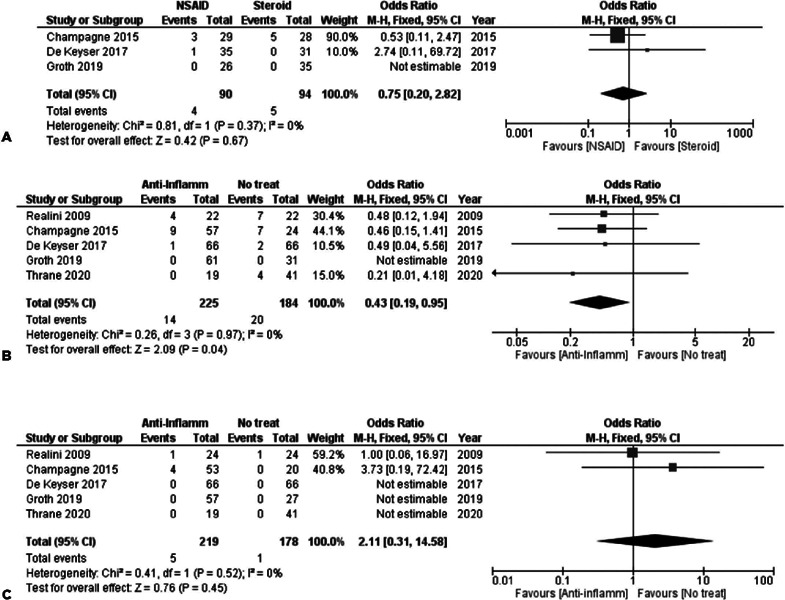
(A) AC inflammation 1 week after SLT-NSAID vs. Steroid. (B) AC inflammation 1 week after SLT-anti-inflammatory vs. No treatment. (C) AC inflammation 4-6 weeks after SLT-anti-inflammatory vs. No treatment.

The results of 3 studies consisting of 90 subjects in the NSAID group and 94 in the steroid group were synthesized. No inferiority was noted in any of the anti-inflammatory treatment 1 week after SLT, with FE OR=0.75, 95% CI = (0.20, 2.82), P_Q_=0.37, I^2^=0% (Figure 6A).

No study revealed any signs of AC reaction during the subsequent follow-up.

#### 2. Anti-Inflammatory versus Placebo/No treatment

As noted with the assessment of IOPs, any groups of NSAIDs or steroids were combined to estimate the overall efficacy of any anti-inflammatory treatment so as to prevent the development of AC inflammation.

Five studies provided data about the first post-SLT week, with a total of 409 subjects. Anti-inflammation treatment was associated with less AC inflammation in the synthesis of the data, with FE OR=0.43, 95% CI = (0.19, 0.95), P_Q_=0.97, I^2^=0% (Figure 6B)]. This correlation did not remain significant 4-6 weeks after SLT, as the results of the synthesis of the same 5 studies suggested [FE OR=2.11, 95% CI = (0.31, 14.58), P_Q_=0.52, I^2^=0% (Figure 6C)].

### C. Analysis per Discomfort/Pain

#### 1. NSAIDs versus Steroids

**Figure 7:**
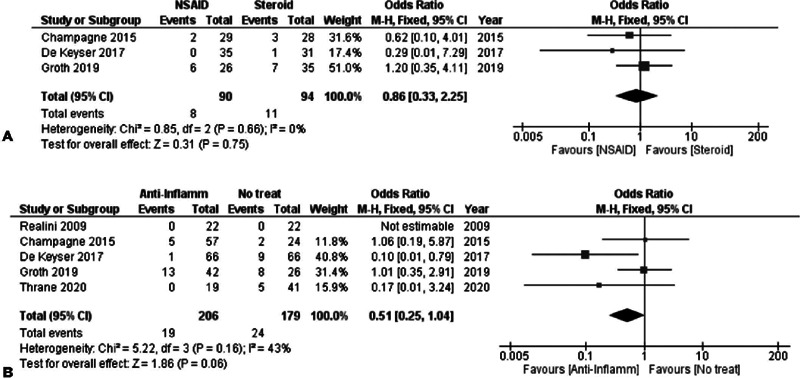
(A) Discomfort/Pain 1 week after SLT-NSAID vs. Steroid. (B) Discomfort/Pain 1 week after SLT-anti-inflammatory vs. No treatment.

Three studies were combined in the analysis of discomfort 1-week post-SLT, including 90 and 94 subjects in the NSAID and steroid groups, respectively. No statistically significant difference was noted between the groups, with a [FE OR=0.86, 95% CI = (0.33, 2.25), P_Q_=0.66, I^2^=0% (Figure 7A)].

#### 2. Anti-Inflammatory versus Placebo/No treatment

Similar to the other relative analyses, any groups of NSAIDs or steroids were combined.

Discomfort after 1 week of SLT was analyzed in 5 studies. By combining these studies, a tendency was noted toward less discomfort for the anti-inflammatory group. A total of 206 and 179 subjects were included in the anti-inflammatory and the no-treatment groups, respectively, providing a FE OR=0.51, 95% CI = (0.25, 1.04), P_Q_=0.16, I^2^=43% (Figure 7b). However, as Q-statistic and I^2^ suggested, the included studies showed moderate heterogeneity.

## DISCUSSION

Glaucoma is a progressive disease that causes irreversible visual loss leading to blindness. First-line treatment for glaucoma included the use of topical eye drops. However, the regular use of preservatives containing eye drops has been associated with several complications, majorly ocular surface diseases^(37,38)^. Moreover, another issue of topical treatment is the questionable compliance of the patients^(39)^. SLT addresses several of these problems. A recent multi-center RCT demonstrated non-inferiority of SLT when compared to eye-drop treatment protocols, as proposed by the European Glaucoma Society and the National Institute for Health and Clinical Excellence^(40)^, in terms of health-related quality-of-life, clinical outcomes, and cost-effectiveness. The LIGHT study conducted by Gazzard et al. revealed that SLT demonstrates an excellent safety profile and is an effective initial treatment for early glaucoma treatment. This evidence-based study provides further evidence for clinicians to consider offering SLT as the first-line treatment for most newly diagnosed glaucoma patients^(41)^. Recently, they also determined that repeat SLT provided effective IOP reduction, which maintained IOP comparable in medication-naive OAG and OHT eyes requiring retreatment^(42)^. However, the use of anti-inflammatory drops after SLT treatment remains a controversial issue. It is therefore crucial to determine the efficacy of anti-inflammatory drops after SLT, mainly because of the theories supporting inflammation to be part of the action mechanism of SLT^(21)^. The present study was designed to enlighten this issue. To the best of our knowledge, no other systematic review and meta-analysis have dealt with this topic in the past.

The literature search in the present study provided 6 relevant RCTs with 3 possible comparisons. All of them included results from three or more studies. A total of 252 eyes receiving anti-inflammatory treatment after SLT and 204 control eyes receiving either placebo or no treatment were included in this meta-analysis. However, the relatively poor quality of some of the studies posed additional challenges to our research.

No significant differences in IOP were noted at different follow-up testing in our meta-analysis. This finding is relevant to both the anti-inflammatory vs. placebo and steroids vs. NSAIDs comparisons, which conforms with other anecdotal studies. Gorla et al. prospectively randomized patients undergoing SLT to either steroids, NSAIDs, or placebo. No statistically significant difference was noted among the 3 treatment arms after 6 months^(43)^. Jinapriya et al. conducted an RCT to assess any difference among prednisolone, ketorolac, and placebo^(31)^. However, the lack of numerical data between the treatment arms does not provide clear evidence of efficacy for either of the treatment. Moreover, a limitation and possible confounder are the relatively low baseline IOPs seen in this RCT, as it has been shown that IOP level affects the results of SLT^(44,45,46)^. The same limitation applies to the study of Rebenitsch et al., who conducted a retrospective chart review to evaluate the administration of loteprednol in patients undergoing 360° SLT. They found that the patients on loteprednol achieved a higher mean reduction in IOP than subjects not treated with loteprednol^(35)^. Rothman et al. presented the results of steroids versus NSAIDs comparison in their RCT at the 2014 meeting of the Association for Research in Vision and Ophthalmology, suggesting that IOP reduction at various points was not associated with the use of steroids or NSAIDs^(47)^. Overall, the finding that IOP reduction is independent of the administration of anti-inflammatory treatment directly disputes the theory proposed in the literature that inflammation is an integral part of the mechanism through which SLT reduces IOP^(48)^.

Regarding AC inflammation, the administration of an anti-inflammatory regimen was associated with fewer inflammatory signs in the first postoperative week. In our comparison, we only assessed the presence of any grade of AC inflammation, irrespective of its severity. However, there may be significant heterogeneity among the studies, as they did not report the exact method for assessing the AC activity. No study used a flareometer to determine the presence of AC inflammation. Only De Keyser et al., Realini et al., and Thrane et al. described the exact method used for assessing AC inflammation^(29,33,34)^. It may be that the other studies were prone to misclassification bias. Jinapriya et al. reported no statistically significant difference among patients using topical anti-inflammation treatment^(31)^. Nevertheless, this study possibly suffers from misclassification bias. While it has been reported that up to 80% of the eyes undergoing SLT may develop signs of anterior uveitis, it is imperative that the use of topical anti-inflammatory medications (either NSAIDs or steroids) may be of benefit, especially in patients under prostaglandin analogs^(49,50,51)^.

Regarding the presence of pain, subjects receiving no anti-inflammatory treatment after SLT were more likely to present with symptoms of pain or discomfort 1-week postoperatively. However, as this symptom was not assessed systemically using a recognized pain scale, it may have led to information bias. Only Champagne et al. assessed pain with a questionnaire^(28)^. Nevertheless, they did not refer to the validity or the reliability of their questionnaire, again leading to a possible recall bias^(28)^.

Several predictors of success for SLT, such as the patient population, type of glaucoma, and SLT-treatment protocol, have been reported in the literature. Subjects with higher baseline IOP and increased angle pigmentation have been reported to benefit more from SLT. Thrane et al., for instance, included 28.3% of patients with PEXG, thus including more pigmented angles^(34)^. Furthermore, De Keyser et al. conducted a study on subjects with normal baseline IOPs to determine whether the anti-inflammatory medications taken after SLT made any significant difference in the IOP-lowering effect of the laser and inflammation^(29)^. Moreover, the SALT study conducted by Groth et al. (the only study that reported a statistically significant difference between anti-inflammatory treatment and placebo) reported an uneven distribution among the 90°, 180°, and 360° SLT protocol groups^(52)^. Only a few studies have examined the efficacy of different SLT protocols, with some of them reporting 360° protocols to provide better outcomes^(53,54,55)^, with others claiming that even 90° or 180° can be effective^(56,57)^. This result may also be affected by the fact that subjects with a wider TM laser received an overall greater amount of energy considering that the SLT energy used is positively correlated with IOP reduction^(58)^.

A review of the relevant literature and analyses of the data revealed that the topical use of anti-inflammatory treatment following SLT is not associated with postoperative IOP measurements. Consequently, the utilization of postoperative medications remains controversial based on the current evidence. However, the administration of a topical anti-inflammatory regimen may be of benefit in cases where very high energy SLT was performed or when extremely pigmented angles were treated as it may prevent the development of anterior uveitis and reduce the occurrence of postoperative pain and discomfort. Larger scale studies including a variety of patients’ glaucoma subtypes and different surgical techniques are warranted in the future to resolve these queries.
